# Expression Analysis in Atlantic Salmon Liver Reveals miRNAs Associated with Smoltification and Seawater Adaptation

**DOI:** 10.3390/biology11050688

**Published:** 2022-04-30

**Authors:** Alice Shwe, Aleksei Krasnov, Tina Visnovska, Sigmund Ramberg, Tone-Kari K. Østbye, Rune Andreassen

**Affiliations:** 1 Department of Life Science and Health, Faculty of Health Sciences, OsloMet-Oslo Metropolitan University, 0167 Oslo, Norway; aliceshw@oslomet.no (A.S.); sigmundr@oslomet.no (S.R.); 2 Nofima (Norwegian Institute of Food, Fisheries and Aquaculture Research), 1430 Ås, Norway; aleksei.krasnov@nofima.no (A.K.); tone-kari.ostbye@nofima.no (T.-K.K.Ø.); 3 Bioinformatics Core Facility, Oslo University Hospital, 0372 Oslo, Norway; martvis@ifi.uio.no

**Keywords:** smoltification, seawater adaptation, microRNAs, small-RNA sequencing, liver, Atlantic salmon, microarray transcriptome

## Abstract

**Simple Summary:**

Smoltification is a developmental process that preadapts Atlantic salmon for a life in seawater. Suboptimal smoltification and poor timing of transfer to seawater is associated with increased mortality. MicroRNAs (miRNAs) are small non-coding genes. They regulate gene expression post-transcriptionally as part of the miRNA induce silencing complex (miRISC) where they guide miRISC to particular mRNAs (target genes). The aim of this study was to identify Atlantic salmon miRNAs expressed in liver that are associated with smoltification and adaptation to seawater as well as to predict their target genes. In total, 62 guide miRNAs were identified, and by their expression patterns they were clustered into three groups. Target gene predictions followed by gene enrichment analysis of the predicted targets indicated that the guide miRNAs were involved in post-transcriptional regulation of important smoltification associated biological processes. Some of these were energy metabolism, protein metabolism and transport, circadian rhythm, stress and immune response. Together, the results indicate that certain miRNAs are involved in the regulation of many of the important changes occurring in the liver during this developmental transition.

**Abstract:**

Optimal smoltification is crucial for normal development, growth, and health of farmed Atlantic salmon in seawater. Here, we characterize miRNA expression in liver to reveal whether miRNAs regulate gene expression during this developmental transition. Expression changes of miRNAs and mRNAs was studied by small-RNA sequencing and microarray analysis, respectively. This revealed 62 differentially expressed guide miRNAs (gDE-miRNAs) that could be divided into three groups with characteristic dynamic expression patterns. Three of miRNA families are known as highly expressed in liver. A rare arm shift was observed during smoltification in the Atlantic salmon-specific novel-ssa-miR-16. The gDE-miRNAs were predicted to target 2804 of the genes revealing expression changes in the microarray analysis. Enrichment analysis revealed that targets were significantly enriched in smoltification-associated biological process groups. These included lipid and cholesterol synthesis, carbohydrate metabolism, protein metabolism and protein transport, immune system genes, circadian rhythm and stress response. The results indicate that gDE-miRNAs may regulate many of the changes associated with this developmental transition in liver. The results pave the way for validation of the predicted target genes and further study of gDE-miRNA and their targets by functional assays.

## 1. Introduction

Atlantic salmon is one of the most successful aquaculture species. The total worldwide production of farmed Atlantic salmon was around 2638 kilotons in 2020 and 1821 kilotons of this was produced in the North Atlantic area [1]. The majority of farmed salmon in the North Atlantic area is produced by Norway and UK (Scotland) (77% and 11%, respectively) [1]. The rapid growth in the salmon aquaculture industry is due to continuous improvement in production practices [2]. However, salmon aquaculture is still facing several challenges including high mortality after sea transfer associated with deficient smoltification and higher susceptibility to infection diseases [3,4,5]. Studies of salmonids smoltification have also revealed changes in expression of genes involved in immune responses [6,7,8,9].

Smoltification (also known as Parr–Smolt transformation) is a complex preparatory developmental process that transforms parr to smolt for a successful life in the marine environment [10,11,12]. Smoltification involves changes in physiology (e.g., increased metabolic rate, increased seawater tolerance and alterations in lipid metabolism) [13], morphology (e.g., smolts acquire silver skin pigmentation and more streamlined body shape) and behavior (e.g., downstream movement and the loss of territorial behavior) [12,14,15]. Smoltification is a highly energy-demanding process and it is associated with decrease in liver glycogen, whole-body lipid content and muscle content in smolt [16,17]. An increase in mitochondrial enzymes activity [18], enzymes of glycolysis, fatty acid and lactate metabolism (i.e., phosphofructokinase (PFK), β-hydroxyacyl-coenzyme A dehydrogenase (HOAD) and lactate dehydrogenase (LDH)) in the liver of Atlantic salmon have also been reported. Elevated levels of thyroid hormones could be responsible for this increase [18,19]. It has also been reported that β-oxidation capacity in liver increased significantly prior to seawater transfer which gives liver an important role in energy production during this period [16]. Citrate synthase activity in liver, gill and kidney was also enhanced during smoltification [18,20] and did not change after seawater transfer [21].

In nature, smolting is stimulated by the increasing day length (photoperiod) in spring and seasonal temperature fluctuations [10]. The aquaculture industry takes advantage of this photoperiod-dependence in the production of seawater-tolerant juvenile salmon [22]. Smolting in salmon aquaculture is artificially achieved by exposing parr reaching a desirable size to a short photoperiod for several weeks and then returning them to continuous light [23]. Optimal smoltification and correct timing of seawater transfer (SWT) are crucial for normal development, growth and health of farmed salmon [24,25]. Various tests for assessing smolt readiness and quality are employed, including salinity tolerance test, seawater challenge hypo-osmoregulatory test (SCHT), mRNA expression and enzymatic activity of gill Na^+^/K^+^-ATPase (NKA) and measurement of hormones involved in smolting [24,26,27,28]. Salinity tolerance test and SCHT are inexpensive and relative quick to perform. The SCHT is used by many commercial hatcheries in Norway and Canada [24]. Studies showed that despite satisfactory reactions to salinity tolerance test and SCHT, some hatchery-reared smolts perform poorly at sea in terms of survival and growth rate compared to their naturally produced smolts [29]. Additionally, gill NKA activity in freshwater at the peak of smolting does not always predict long-term growth in seawater [30].

In recent years, incomplete smoltification is reported as one of the most important causes of mortality and contributor to disease development in the period following sea transfer in Norwegian salmon farms [5,25]. According to the annual summary of fish health in Norway [25] from the Norwegian Veterinary Institute, 52.1 million of 290 million smolts transferred to sea died prior to harvest in 2020. The high mortality rate of farmed salmon in the period following sea transfer represents a major fish welfare problem as well as a major economic loss for the aquaculture industry. Better measures for optimal smoltification and transfer to sea at the correct time and good follow up during the first period in seawater are required to reduce mortalities and improve welfare of farmed salmon [25]. Research on smoltification in the past 30 years was particularly directed to gain a better understanding of optimal timing for transfer of smoltified juveniles into ocean net pens [3,26]. In recent years, more studies of gene expression associated with smoltification have been carried out to better understand this developmental transition as well as to search for new biomarkers [7,22,31].

MicroRNAs (miRNAs) are single-stranded, non-coding RNAs (typically ~22 nucleotides in length) that regulate a large variety of biological processes at the post-transcriptional level [32,33]. Mature miRNAs are processed by a cascade of several nuclear and cytoplasmic enzymatic processing steps [34]. Most miRNAs are transcribed into long primary miRNAs (pri-miRNAs) that are processed further into hairpin-structured precursor miRNAs (pre-miRNAs) and finally short miRNA duplexes [35]. At the final step, one of the strands of the miRNA duplex (termed as “mature miRNA” or “guide miRNA”) is loaded into and retained in Argonaute (AGO) proteins, forming the miRNA-induced silencing complex (miRISC). The other strand of the miRNA duplex (termed as “passenger miRNA”) is released from AGO proteins and degraded. The guide miRNAs direct the miRISCs to their target messenger RNAs (mRNAs), usually by binding partially to the 3′-untranslated region (3′-UTR) of the target transcripts. This leads to translational repression or degradation of target mRNAs [36]. The guide miRNA may originate from the 5′ end of the pre-miRNA (referred to as “5p”) or the 3′end of the pre-miRNA (referred to as “3p”) [34,37]. Atlantic salmon miRNAs are among the best characterized in teleost [38]. Due to the salmonid specific genome duplication [39,40], the number of miRNA gene families is larger in salmonids than in any other teleost [38,41]. Recently, the transcriptomes from liver, head kidney and gills from different stages of the smoltification process were characterized by full-length error corrected mRNA sequencing. This provided a catalog of genes and splice variants that are expressed in liver during this developmental process [42]. Furthermore, the 3′UTRs from the full-length sequenced mRNAs were analyzed regarding their potential as miRNA targets. This resulted in a comprehensive resource of predicted mRNA targets for any of the mature miRNAs in Atlantic salmon [43].

The miRNA expression analysis often shows that one of the mature miRNAs appears as the highly expressed one compared to the other mature miRNA processed from same precursor. This is a consistent pattern shown in several studies [38,41]. The explanation to this difference in abundance of the two mature miRNAs from one precursor is that the more abundant one is the biologically important guide miRNA incorporated into miRISC while the other one with the much lower abundancy, is the passenger miRNAs that are degraded [37]. Here, in cases where there is a large difference in abundance between miRNAs from same precursor, we refer to the abundant mature miRNAs (10 times higher read counts than its corresponding mature from same precursor) as the biologically important guide miRNAs. Previous mammalian studies have characterized some miRNAs that were highly expressed in liver [44,45], suggesting that they are important regulators in liver development [46], liver homeostasis, ion metabolism and lipid, and cholesterol biosynthesis [47,48,49]. Studies in teleost fish have also reported that miRNAs are involved in immune response [50,51,52] and response to environmental stimuli [53]. A study of hepatic miRNAs in rainbow trout and farmed carp indicated that miRNAs are involved in hepatic energy metabolism [54,55] while a study in Atlantic salmon revealed several miRNAs associated with lipid metabolism in liver [56]. Furthermore, miR-122-5p, miR-8163-3p, miR-148-5p and miR-101-3p have been reported as highly expressed in the liver of marine teleost, indicating that they serve a common important function in this organ [38,57].

Our recent study conducted in the head kidney of Atlantic salmon indicated that miRNAs are involved in post-transcriptional regulation of gene expression during smoltification and adaptation to seawater in this organ [58]. Gene ontology analysis of the predicted target genes showed that they were enriched in head kidney specific biological processes associated with smoltification and seawater adaptation [58]. So far, similar studies have not been carried out in liver, an organ that plays an important role in storage (lipids, carbohydrates, vitamin A and iron) and detoxification in teleost [59]. In addition, the liver has a central position in amino acid and carbohydrate metabolism and in the synthesis and export of many proteins [60].

The aim of the present study was to characterize miRNA expression changes associated with smoltification and adaptation to seawater in the first month following seawater transfer. Investigating the post-transcriptional interaction between miRNAs and their predicted target mRNAs during this critical period of Atlantic salmon life may provide a better understanding of how the fine-tuning of gene expression in liver may help in facilitating this developmental transition.

## 2. Materials and Methods

### 2.1. Experimental Fish Trial and Samplings

The experiment was carried out at the Nofima’s Research Station for Sustainable Aquaculture (Sunndalsøre, Norway) in accordance with the Guidelines of the EU-legislation (2010/63/EU), as well as with the Norwegian legislation on animal experimentation. The experimental fish were not exposed to any pain or distress. They were solely killed for the use of their tissues in this project and, thus, approval from the Norwegian Food Safety Authority was not required.

The experimental fish were from SalmonBreed commercial strain SalmonBreed-Model SB-Optimal. The most important characteristic of this strain is good growth combined with a balanced weighting for health properties through family selection. A total of 70 fish were selected for this experiment. The fish were kept in one tank supplied with running water throughout the experimental period and they were fed commercial dry feed (Skretting, Norway). The fish used in this study were the same experimental fish as described in Shwe et al. [58]. From the start of feeding, the fish were kept in freshwater with an average water temperature of 13 °C and 24 h continuous light. The average water temperature was then dropped to 8 °C 2 weeks before the start of smoltification process. The initial smoltification process started by decreasing daylight from 24 h to 12 h and increasing water temperature from 8 °C to 13 °C for 5 days, followed by 12 °C for 41 days. Subsequently, the daylight was increased to 24 h and the water temperature lowered to 8 °C for the final stage of smoltification (Table 1). The seawater challenge test was performed once a week in the last 3 weeks before SWT using a salinity of 35‰. Seawater challenge test, blood plasma ions (Cl^−^, Na^+^ and Mg^2+^) level and the change to silvery skin color indicated that the experimental fish were smoltified 81 days after onset of the experiment. The smoltified smolts with average weight 72.4 ± 8.7 g were then transferred to seawater. The average weight at 1-week post-SWT and one-month post-SWT was 63.2 ± 8.5 g and 98.4 ± 14.9, respectively. No mortality of smolts was observed during smoltification or after SWT.

The liver samples were collected at six time points (Table 2), T1: parr, 1 day prior to light treatment, T2: halfway through light treatment at the change from 24 h to 12 h light and temperature to 12 °C (47 days post-onset of light treatment (POL)), T3: three quarters into the light treatment period (67 days POL), T4: smolt, 1 day prior to SWT (81 days POL), T5: 1 week after SWT (88 days POL), and T6: 1 month after SWT (111 days POL). The experimental conditions such as day light, water temperature, average weight of experimental fish and water type at each sampling points are provided in Table 2. Ten fish were euthanized with an overdose of anesthetic metacain (MS-222; 0.1 g/L) and killed by a blow to the head prior to weighing and sampling at each time point. The collected liver samples were frozen immediately in liquid hydrogen and stored at −80 °C. Seven fish from each time points were used for RNA extraction and small RNA sequencing, while RNA from five of these fish was also used for microarray analysis.

### 2.2. Total RNA Extraction for Sequencing and Microarray Analysis

Total RNA was isolated using the mirVanaTM miRNA Isolation Kit (Ambion, Life Technologies, Carlsbad, CA, USA) according to the manufacturer’s protocol. The RNA quality and quantity were determined using NanoDropTM1000 Spectrophotometer (Nanodrop ND-1000, Thermo Fisher Scientific, Wilmington, DE, USA). The integrity of total RNA (RIN value) was measured using the Agilent 2100 Bioanalyzer in combination with an Agilent 6000 Nano Chip (Agilent Technologies, Santa Clara, CA, USA). Extracted total RNA was stored at −80 °C.

### 2.3. Small-RNA Library Preparation and Sequencing

Library construction and sequencing of 42 liver samples were carried out at the Norwegian High-Throughput Sequencing Centre (NSC; Oslo, Norway). Seven liver samples from each of the time points T1-T6 were selected for sequencing. The NEBnext^®^ multiplex small RNA Library Prep Set (New England Biolabs, Inc., Ipswich, MA, USA) was used to construct libraries for 42 liver samples in accordance with manufacturers protocol. One µg total RNA from each sample were used as input for preparation of the libraries followed by 5′ and 3′ adapter ligation, reverse transcription, PCR amplification and size selection of 140–150 bp fragments using 6% polyacrylamide gel. Sequencing was performed on a NextSeq 500 from Illumina (Illumina, Inc, San Diego, CA, USA), producing 75 bp single end reads. All sequenced samples have been submitted to the NCBI Sequence Read Archieve Centre (SRA) (https://www.ncbi.nlm.nih.gov/sra accessed 20 April 2022) with accession bioproject number PRJNA665200 and will automatically be released by NCBI at the publication of this study.

### 2.4. Processing of Small-RNA Reads and DESeq2 Expression Analysis

Data processing and quality control of small-RNA reads were performed according to the procedure described in Shwe et al. [58]. The quality of raw reads was checked using FASTQC software (v.0.11.8). The raw reads that passed quality control were trimmed using Cutadapt (v.2.3) Python package (v.3.7.3) [61]. Trimmed reads were processed further by size filtering to discard all reads that were shorter than 18 nucleotides (nts) or longer than 25 nts. An additional FASTQC analysis was performed to make sure that there were no adapter sequences or poor-quality reads in our final data set of clean reads.

Two samples from smoltified fish (T4) and saltwater-adapted fish (T6) were used in mirDeep2 analysis (v.0.0.7) [62] for miRNA discovery as described in Woldemariam et al. [38], but no Atlantic salmon miRNAs other than those already described in the Atlantic salmon miRNAome [38] were discovered. Subsequently, clean reads were aligned to the reference index of all known *Salmo salar* mature miRNAs [38] using STAR aligner software with default parameters except modified with parameter—alignIntronMax 1 (v.2.5.2b) [63]. The output files of STAR alignment (BAM format) were processed further in R-studio using the feature Counts function from Rsubread package (v.1.34.2) to produce matrices [64]. These count tables were used as input in the DESeq2 R package (v.1.24.0) for miRNA differential expression analysis by comparing each of the time points T2, T3, T4, T5 and T6 with T1. DESeq2 performs an internal normalization by estimating the size factor for each sample. The size factor is estimated by first calculating geometric mean for each gene across all samples. The counts for a gene in each sample is then divided by this geometric mean. The median of these ratios in a sample corresponds to the size factor for that sample [65].

All miRNAs with log2 fold-change ≤−1.0 or ≥1.0, Benjamini-Hochberg adjusted *p*-value ≤ 0.05 and with average normalized read counts > 30 in at least at one comparison were defined as differentially expressed miRNAs (DE-miRNAs). Subsequently, read counts of DE-miRNAs originating from the same precursor were compared. If one showed >10 times more reads than the other mature from the same precursor, this one was assumed to be the biologically active guide miRNA and subsequently used in the in silico target analysis and gene enrichment analysis. Additional unsupervised hierarchical clustering with complete linkage and spearman correlation was performed with DE-miRNA log2-fold changes as input using *hclust* function from the stats package (v.3.6.1) in R. Heatmap2 from R-package gplots (v.3.0.1.1) was used to plot heatmaps of DE-miRNAs grouped by the hierarchical clustering analysis.

### 2.5. Microarray Analysis

The expression profiling of differentially expressed mRNAs (DE-mRNAs) in the liver of Atlantic salmon was performed at NOFIMA (Ås, Norway) using 44 k DNA oligonucleotide microarray containing 60-mer probes to protein coding genes (Salgeno-2, GPL28080). The oligonucleotide microarray for Atlantic salmon were designed at Nofima and annotated with bioinformatics package STARS [66]. Microarrays were manufactured by Agilent Technologies (Inc., Cedar Creek, TX, USA), and the reagents and equipment were from the same source. One-color hybridization was used, and each sample was analyzed with separate array. Microarray was performed on 28 of the 42 liver samples selected for small-RNA sequencing, representing six time points (T1–T6), with five fish per time point except T5 and T6 where four fish were analyzed.

Total RNA (220 ng) from each sample was used as input for cDNA synthesis, amplification and Cy3 labeling of cRNA using a LowInput QuickAmp Labeling Kit according to the manufacturer’s protocol. The labeled/amplified cRNA were purified using Qiagen’s RNeasy Mini Kit (QIAGEN group, Hilden, Germany). The quantity and quality of the purified cRNA was assessed by NanoDropTM1000 Spectrophometer (Nanodrop ND-1000, Thermo Fisher Scientific, Wilmington, DE, USA). Cy3-labeled cRNA (1650 ng) was used as input to prepare the hybridization mix for each sample using Gene Expression Hybridization Kit. Slides were hybridized in oven (17 h, 65 C, rotation speed 0.01 g). The hybridized slides were washed and scanned with SureScan Microarray Scanner (Agilent Technologies, Santa Clara, CA, USA). Nofima’s bioinformatic package STARS [66] was used for subsequent data processing of mRNA array data. Differential expression analyses were carried out by comparing each of the time points (T2–T6) with T1. The transcripts/mRNAs with log2 fold-changes ≤−0.80 or ≥−0.80 and *p* < 0.05 (t-test) were defined as significantly changed and termed differentially expressed mRNAs (DE-mRNAs).

Enrichment analysis of DE-mRNAs was performed as described in Krasnov et al. [67] by comparing the numbers of DE-mRNAs per functional category using GO and STARS annotation data sets. Additional pathway analysis was carried out using KEGG annotation data set. Functional categories or pathways with ratio ≥2 and Yates’ corrected chi-square (*p* ≤ 0.05) were defined as overrepresented in DE-mRNA enrichment analysis.

### 2.6. In Silico Target Gene Predictions and Enrichment Analysis of Predicted miRNA Targets

Prediction of guide DE-miRNA targets was carried out against the DE-mRNAs. Firstly, to provide 3′UTR sequences for in silico prediction, probe sequences of the 5708 DE-mRNAs on the microarray were utilized to identify matching transcripts in the Atlantic salmon full-length (FL) mRNA transcriptome [42]. Analysis was performed with the BLASTN tool in the BLAST+ package (v.2.9.0+) [68]. Match criteria of 90% sequence identity and 91% query coverage were used to classify an alignment of a probe sequence as identifying its matching transcript in the FL-transcriptome. The set of FL-transcripts that were classified as matches to at least one DE-mRNA was used as input in the MicroSalmon GitHub repository (http://github.com/AndreassenLab/MicroSalmon/ accessed 20 April 2022) [43]. This produced a list of transcripts that were identified both as being DE-mRNAs and predicted as targets of the gDE-miRNAs revealed in this study (FL-targets).

The gene symbols of FL-targets were retrieved from both MicroSalmon [43] and the Universal Protein Resource (UniProt) (https://beta.uniprot.org/ accessed 20 April 2022) using the FL-target transcripts annotation information as input. Gene ontology enrichment analysis was performed using PANTHER Overrepresentation Test (version 16.0) (http://pantherdb.org/ accessed 20 April 2022) [69]. *Homo sapiens* was chosen as reference gene list for the enrichment analysis as this is the most complete functional annotated database while teleost is rather incompletely annotated. The annotation data sets GO biological process complete and Reactome pathways were used to identify enriched biological processes (BP) and gene pathways associated with DE-miRNA targets, respectively. Fold enrichment (FE) ≥2 and Fisher’s Exact test with False Discovery Rate (FDR) less than 0.05 as calculated by the Benjamini–Hochberg procedure were used as thresholds in the enrichment analysis. Subclasses that were related to the same functional category or pathway category were grouped together in the PANTHER analysis and sorted by most specific subclasses. The grouped outputs also show the related biological processes ranked from general to specific biological processes (see Supplementary Files in Section 3.6).

## 3. Results

### 3.1. RNA Library Preparation and Small RNA Sequencing

Total RNA from 42 liver samples collected before, during smoltification and post-SWT was successfully extracted, and all samples were subsequently small-RNA sequenced. The number of raw reads obtained from small-RNA sequencing of liver samples (n = 42) ranged from 5.7 to 12.3 million. The quality-filtered (Phred score >32), adapter-trimmed and size-filtered reads for each sample ranged from 3.3 million to 8.4 million. The clean reads uniquely mapped as mature *Salmo salar* miRNAs in each sample ranged from 49.9 to 79.6%. An overview of all samples including their RNA concentration, RIN value, read numbers, reads uniquely mapped as mature miRNAs and the SRA accession numbers are given in Table S1.

### 3.2. miRNAs with Differential Expression Changes in Liver during Smoltification and Post SWT

To identify DE-miRNAs, we compared the miRNA expression before smoltification (parr) (T1) against the ongoing smoltification period (T2, T3), smolt (T4) and samples from post-SWT (T5 and T6). An overview of conditions at different sampling points is given in Table 1. A total of 88 miRNAs (Benjamini–Hochberg adjusted *p*-value ≤ 0.05, log2 fold-change ≤−1.0 or ≥1.0 and average normalized read counts >30) belonging to 47 miRNA families were differentially expressed relative to T1 on at least one of the five time points. The relative expression changes of the 88 DE-miRNAs at each timepoint and the mature miRNA sequences of each of the DE-miRNAs is given in Table S2. A heatmap illustrating changes in expression pattern of all 88 DE-miRNAs at all time points is given in Figure S1.

**Figure 1:**
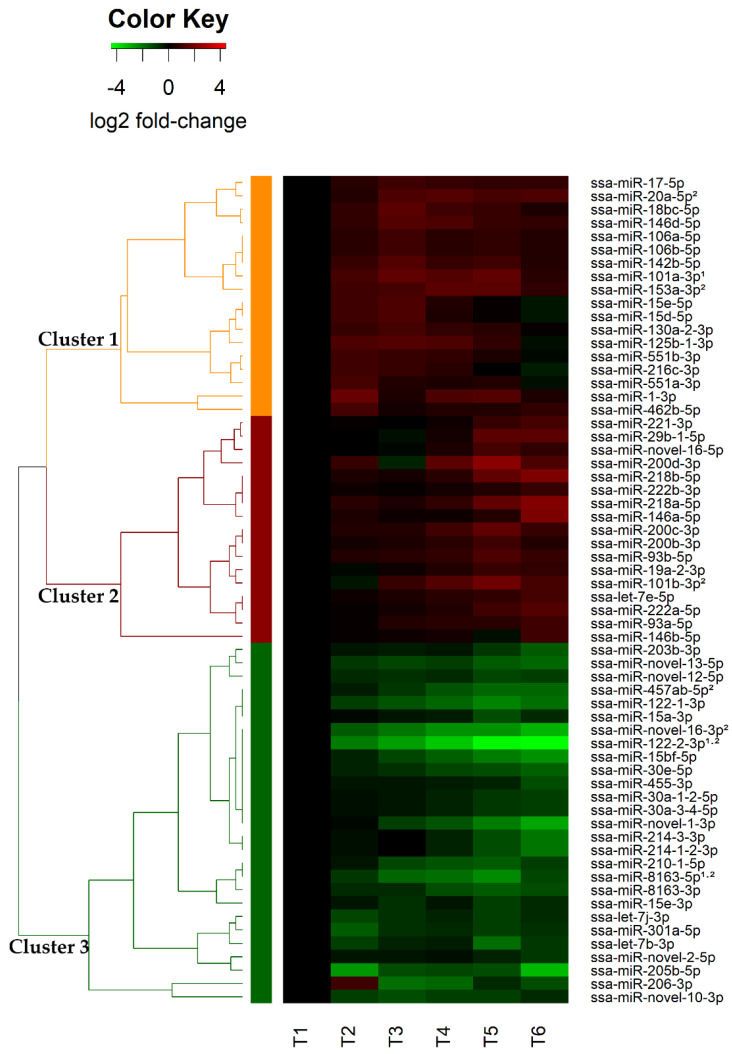
Heatmap and hierarchical clustering of the 62 guide DE-miRNAs. Each row represents a miRNA, and each column represents the expression changes at each time points relative to T1 (pre-smolt, one day before smoltification). T2–T4 and T5–T6 are relative expression changes during smoltification period and post-SWT period, respectively. The dendrogram and the row side colors on the left show the three major clusters of DE-miRNAs (Cluster 1-orange, Cluster 2-red, Cluster 3-green). The direction of expression changes in terms of log2 fold-change is illustrated by the color key above the heatmap. The annotation (1) indicates liver-specific miRNAs and (2) indicates some miRNAs with large changes from T1 to T4 (smoltified fish).

There were 62 among these 88 that were identified as guide DE-miRNAs (gDE-miRNAs), meaning that they were the highly abundant mature miRNA of the two miRNAs originated from the same precursor or both mature miRNAs if present in similar amounts (see definition in introduction and methods). Read counts of the gDE-miRNAs are given in Table S3. The annotated gDE-miRNAs in this study agreed with the previously characterized differences in abundance between miRNAs from same precursor [38,41]. Hierarchical clustering analysis of these 62 gDE-miRNAs revealed 3 major clusters. The results are illustrated in the heatmap in Figure 1.

Cluster 1 consisted of 18 gDE-miRNAs belonging to 15 miRNA families (Table 3). This cluster was characterized by larger increases in miRNA expression occurring during smoltification including some gDE-miRNAs with modest changes 1 week post-SWT (Figure 1). The second cluster consisted of 17 gDE-miRNAs from 11 miRNA families including ssa-miR-novel-16 which has so far only been discovered in Atlantic salmon (Table 3). The expression pattern common to cluster 2 gDE-miRNAs was a small upregulation of their expression during smoltification (T2–T4) that continued in a larger increase and peaked post-SWT (T5–T6). The gDE-miRNAs included in cluster 3 were those characterized by a decrease in their expression relative to T1. The expression of some miRNAs in this cluster decreased gradually from T1 to T6 while others decreased at later time points (Figure 1). Common to all these, except for ssa-miR-206-3p, were that the downregulation peaked after SWT. There were 27 miRNAs in this cluster belonging to 19 miRNA families. Notably, six of these miRNAs have only been discovered in Atlantic salmon (ssa-miR-novel-1, ssa-miR-novel-2-5p, ssa-miR-novel-10-3p, ssa-miR-novel-12-5p, ssa-miR-novel-13-5p and ssa-miR-novel-16-3p) (Table 3).

### 3.3. Liver Specific DE-miRNAs, ARM-SHIFT and Potential Biomarker miRNAs

Three of the DE-miRNAs (ssa-miR-101a-3p, ssa-miR-122-2-3p and ssa-miR-8163-5p, Table 3) are among those highly expressed in the liver of teleost fish and are assumed to have liver-specific functions [38,57]. The expression of the liver-specific ssa-miR-101a-3p (cluster 1, Figure 1) showed an increase during smoltification and 1 week post-SWT followed by a slight decrease in expression 1-month post-SWT. The liver-specific ssa-miR-122-2-3p (cluster 3, Figure 1), on the other hand, showed a decrease in expression during smoltification followed by an even larger decrease in expression after SWT. The liver-enriched miRNA, ssa-miR-8163-5p, also belonged to cluster 3. This miRNA was characterized by a decrease in expression but differed from ssa-miR-122-2-3p by peaking 1 week post-SWT (T5, Figure 1).

Ssa-miR-novel-16-5p increased in expression during smoltification and post-SWT while ssa-miR-novel-16-3p decreased in its expression across all time points. Interestingly, the mature 5p and 3p showed a significantly inverse correlation relationship from T1 to T6 with Spearman’s rho coefficient of −0.83 and *p* = 0.04. Taken together, this revealed that there was a change of arm dominance of the mature miRNAs processed from the miR-novel-16 precursor. The change was from 3p being the major expressed mature miRNA at pre-smolt stage (433 normalized read counts at T1, 69 normalized read counts at T6) to 5p being the major expressed miRNA post-SWT (85 normalized read counts at T1, 197 normalized read counts at T6). Such changes are referred to as arm shifts. In this case, the arm shift was occurring gradually over the experimental timepoints measured.

The expression changes of some gDE-miRNAs (miR-20a-5p, miR-153a-3p, miR-101b-3p, miR-457ab-5p, miR-novel-16-3p, miR-122-2-3p and miR-8163-5p) were rather large from T1 to T4 (smoltified fish, 1 day prior to SWT) (Figure 1), indicating potential biomarkers for identifying smoltified fish. On the other hand, eight miRNAs (miR-140-5p, miR-107-3p, miR-103-3p, miR-130a-3p, miR-148a-3p, miR-199a-3p, miR-22a-3p and miR-21a-5p) showed stable expression across all time points. These eight miRNAs would be suitable reference miRNAs in RT-qPCR analysis of miRNAs changing expression over this developmental transition. Three of these miRNAs miR-183, miR-140 and miR-107 have previously been validated as stable miRNAs suitable as references in RT-qPCR analysis [57].

### 3.4. Identification of DE-mRNAs and Enrichment Analysis of DE-mRNAs

The same liver samples and time-points used for characterization of miRNA expression were used in the microarray analysis. This analysis revealed 5708 mRNAs that were differentially expressed relative to T1 on at least one of the time points compared. The expression changes of all DE-mRNAs over the experimental period and p-values are provided in Table S4.

To identify whether these DE-mRNAs were associated with particular biological processes or gene pathways, we performed enrichment analysis. STARS and GO annotation data sets were used for finding enriched functional categories and KEGG was used for gene pathways enrichment analysis. Enriched biological categories and pathways with a ratio ≥ 2 and *p* ≤ 0.05 are given in Table S5. Several biological processes are associated with smoltification in liver, including glycogen metabolic process [16], fatty acid metabolism, lactate regulation [19], stress response [67], mitochondrial matrix and energy metabolism [18] were overrepresented. Immune-related functional groups have previously been reported in head kidney [7,58]. Additionally, in the liver samples investigated here, there were several immune-related functional groups that were enriched (immune antigen presentation and immune complement).

### 3.5. In Silico Prediction Revealed 2804 gDE-miRNA Target Genes

A common approach to understanding the regulatory role of the miRNAs showing differential expression is to predict their target genes. We, therefore, carried out in silico target prediction with the 62 gDE-miRNAs (Figure 1 and Table 3) against all the 5708 DE-mRNAs identified by microarray analysis (Section 3.4).

The probe sequences from the microarray were used to identify their matching FL-transcripts (described in Section 2.6). Applying the 3′UTRs from these transcripts as input showed that there were DE-mRNAs from 4188 unique loci that were predicted as targets of the gDE-miRNAs. The number of genes were further reduced, as many of the genes from the 4188 loci were duplicates (isoforms or paralogs). The final target gene set consisted of 2804 different genes. A complete overview of predicted mRNAs targets along with their targeting miRNAs, gene names and symbols, transcript accession numbers and GO terms are given in Table S6.

### 3.6. Enriched Biological Processes and Pathways Associated with the Predicted Target Genes

**Figure 2:**
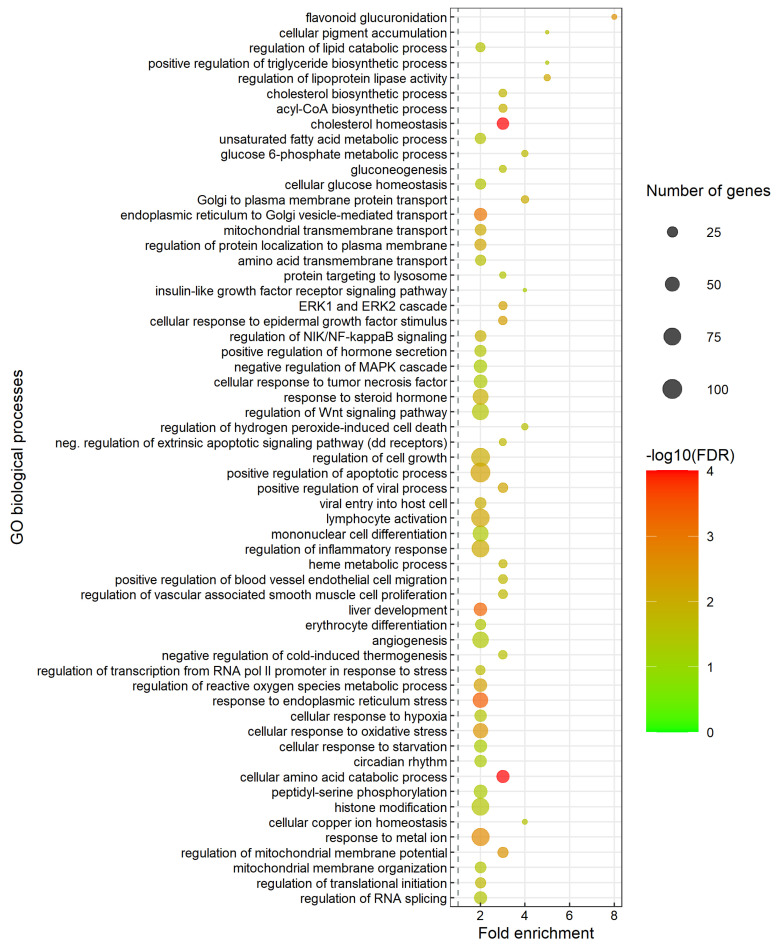
Significantly overrepresented biological processes in the predicted target genes of gDE-miRNAs dataset. The specific and representative subclasses of biological processes are shown here while the complete results from analysis of enriched biological processes are given in Table S8. The dot size indicates the number of DE-miRNA target genes associated with the process and the dot color indicates the significance of the enrichment (−log10 (FDR-corrected P-values)). The vertical grey dashed line represents a fold enrichment of 1.

**Figure 3:**
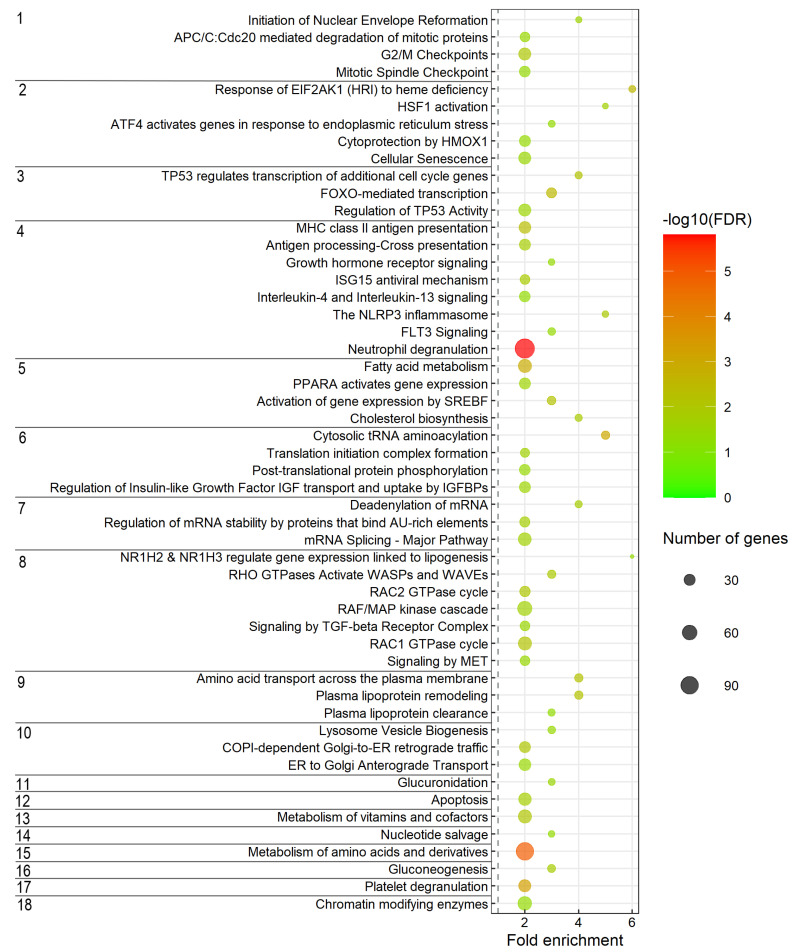
Enriched gene pathways associated with predicted target genes. The specific and representative subclasses of pathways are shown on the *y*-axis while the complete results from the analysis of enriched Reactome pathways are given in Table S9. The enriched pathways were grouped into categories and each category were assigned a number where each number represents the following categories, 1. Cell cycle, 2. Cellular responses to stress, 3. Gene expression (Transcription), 4. Immune system, 5. Metabolism of lipids, 6. Metabolism of proteins, 7. Metabolism of RNA, 8. Signal Transduction, 9. Transport of small molecules, 10. Vesicle-mediated transport, 11. Biological oxidations, 12. Programmed cell death, 13. Metabolism of vitamins and cofactors, 14. Metabolism of nucleotides, 15. Metabolism of amino acids and derivatives, 16. Metabolism of carbohydrates, 17. Hemostasis, 18. Chromatin organization). The dot size indicates the number of DE-miRNA target genes associated with the pathway and the dot color indicates the significance of the enrichment (−log10 (FDR-corrected *p*-values). The vertical grey dashed line represents a fold enrichment of 1.

Enrichment analysis was carried out to determine enriched biological processes and gene pathways associated with the 2804 predicted target genes (Table S7). The specific subclasses of enriched biological processes are shown in Figure 2 while Figure 3 shows the enriched gene pathways. The complete outputs from gene ontology (GO) enrichment analysis with term biological process (BP) and gene pathways enrichment analysis are provided in Tables S8 and S9, respectively.

The enriched biological processes included carbohydrate biosynthesis, amino acid metabolism, lipid biosynthesis, steroid metabolic process (cholesterol biosynthesis), apoptotic process and protein transport (Figure 2). Biological processes related to response to environmental stimuli that are associated with the developmental changes during smoltification were also enriched. This included response to stress, external stimuli (circadian rhythm) and organ developmental processes (liver development and angiogenesis).

The gene pathways analysis (Figure 3), also revealed enriched pathways associated with those revealed in the enrichment analysis of biological processes. Interestingly, several pathways related to immune-system-like MHC class II antigen presentation and antigen-processing–cross presentation, immune system by Cytokine signaling-like growth hormone receptor signaling, ISG15 antiviral mechanism, Interleukin-4 and Interleukin-13 signaling including the NLRP3 inflammasome, FLT3 signaling and Neutrophil degranulation were also revealed in the gene pathways enrichment analysis (Figure 3). The other significantly enriched pathways were mainly related to cell cycle, cellular responses to stress, transcription, signal transduction, small molecules and vesicle-mediated transport, biological oxidation, cell death, hemostasis and metabolism (lipids, proteins, RNA, nucleotides, amino acids, derivates, vitamins and cofactors).

## 4. Discussion

### 4.1. Differential Expression of 62 Guide miRNAs Indicates That They Are Involved in Post-Transcriptional Gene Regulation during Smoltification and Seawater Adaptation

The differential expression analysis of miRNAs investigated here allowed us to identify 88 miRNAs that changed their expression during smoltification. Sixty-two of them were annotated as the biologically important guide miRNAs (Table 3) by examining their level of average normalized read counts compared to their corresponding mature miRNAs originated from the same precursor (Table S3). In a few cases, DE-miRNAs from the same precursor (e.g., ssa-miR-8163-3p and ssa-miR-8163-5p, Table S3) revealed similar mature miRNA read counts. In cases like this, both mature miRNAs were regarded as biologically important gDE-miRNAs. The hierarchical analysis of the 62 gDE-miRNAs also revealed that DE-miRNAs in the same family showed similar expression pattern. Consequently, same family DE-miRNAs usually clustered together as either upregulated or downregulated. This is also consistent with expected results for mature miRNAs from same family having the same regulatory function [70].

Among the gDE-miRNAs, there were three miRNA families (miRNA-101, miRNA-122 and miRNA-8163) that have previously been reported as liver-specific in teleost [38,57]. Two of these, miR-101a-3p and ssa-miR-122-2-3p, were predicted to target genes involved in fatty acid metabolism (e.g., FAM3A-like protein, non-specific lipid-transfer protein and diacylglycerol O-acyltransferase 2-like) and xenobiotic glucuronidation (e.g., UDP-glucuronosyltransferase-like isoform and multidrug resistance-associated protein 4-like), respectively. The third one, miR-8163-5p, was predicted to target genes that are associated with liver regeneration (e.g., hepatocyte growth factor-like protein and hepatocyte nuclear factor 3-beta-like) [71]. Another predicted target gene of miR-8163-5p was insulin-like growth factor-binding protein 1 (IGFBP-1), a regulator that is mainly produced in liver [72]. IGFBP-1 is known to bind to insulin-like growth factor (IGF-I) [73], an important regulator for smoltification [26]. In addition, fatty acid synthase was also a predicted target of miR-8163-5p. This is a housekeeping protein that is involved in production of fat for storage when nutrients are present in excess in liver [74]. Altogether, these observations were in agreement with these three liver-specific miRNAs being involved in regulation of biological processes associated with liver-specific functions affected by smoltification and adaptation to seawater.

When comparing results from differential expression analysis of miRNAs in head kidney (HK) [58] and liver (this study) sampled from the same experimental fish group, there were 12 miRNAs (ssa-let-7b-3p, ssa-miR-1-3p, ssa-miR-29b-1-5p, ssa-miR-125b-1-3p, ssa-miR-146a-5p, ssa-miR-153a-3p, ssa-miR-200b-3p, ssa-miR-205b-5p, ssa-miR-218a-5p, ssa-miR-301a-5p, ssa-miR-novel-2-5p and ssa-miR-novel-12-5p) that were gDE-miRNAs in both head kidney and liver. Four of these miRNAs (ssa-miR-29b-1-5p, ssa-miR-146a-5p, ssa-miR-153a-3p and ssa-miR-218a-5p) showed an increasing expression during smoltification and SWT while six miRNAs (ssa-miR-1-3p, ssa-let-7b-3p, ssa-miR-125b-1-3p, ssa-miR-205b-5p, ssa-miR-novel-2-5p and ssa-miR-novel-12-5p) showed a decreasing expression during smoltification and SWT. The fact that they revealed same expression profiles in different organs indicates that they are involved in regulation of basic cellular homeostasis, most likely not associated with organ specific functions in kidney or liver. The two remaining miRNAs (ssa-miR-200b-3p and ssa-miR-301a-5p) revealed differences in expression profiles between liver and HK, showing an increased expression level in liver and a decreased expression level in HK.

One DE-miRNA, miR-novel-16 revealed an arm-shift gradually changing from a dominant 3p at pre-smolt stage to a dominant 5p at post-SWT stage. miRNA arm shifting is a change in the preferential enrichment of 5′ or 3′ mature miRNA from a precursor. This has previously been reported as highly dynamic, tissue-specific and could also be time-dependent [75,76]. This finding indicates that both miR-novel-16-3p and miR-novel-16-5p were biologically functional, but the preferences of 3p or 5p as guide miRNAs depended on the developmental stage of the fish.

Seven miRNAs showed rather large changes from T1 (parr) to T4 (smolt) (miR-20a-5p, miR-153a-3p, miR-101b-3p, miR-457ab-5p, miR-novel-16-3p, miR-122-2-3p and miR-8163-5p). Three of these miRNAs (miR-20a-5p, miR-8163-5p and miR-novel-16-3p) predicted to target genes (Na^+^,K^+^-ATPase(NKA) α- and β-subunit isoforms) that are often considered as indicator of smolt development in gill [24]. These gDE-miRNAs with large expression changes from T1 (parr) to T4 (smolt) point themselves out as potential novel biomarkers for assessing smoltification status in Atlantic salmon. An RT-qPCR assay applying these as biomarkers would require reference miRNAs that are stably expressed. Eight miRNAs (miR-140-5p, miR-107-3p, miR-103-3p, miR-130a-3p, miR-148a-3p, miR-199a-3p, miR-22a-3p and miR-21a-5p) could fit as such reference genes since their expression were stable across all the six time points. In particular, miR-183, miR-140 and miR-107 are good candidates, as they previously have been validated as stable and worked well (efficiency and specificity) in RT-qPCR assays [57]. Although appearing as promising biomarkers, they need to be further validated experimentally. In summary, three different expression profiles of gDE-miRNAs identified here show that some miRNAs expressed in liver are associated with smoltification and seawater adaptation.

### 4.2. Enriched Biological Processes and Pathways Associated with Predicted DE-miRNA Targets

The enriched biological categories observed in the microarray analysis were, in general, in agreement with findings observed in previous studies of differentially expressed genes [7,58] and physiological and biochemical changes [16,18,19] associated with smoltification. The DE-mRNAs discovered in the microarray analysis were used as input in the in silico target prediction analysis. The predictions were carried out in this manner to make the predictions relevant to genes important in this developmental transition. Aiming to further minimize false positives, only predicted target genes supported by RNAhybrid and at least two of the prediction tools (PITA, miRanda, TargetSpy) were included as putative target genes [43]. Applying this strategy, the in silico target predictions revealed 2804 unique genes as potential targets.

Enrichment analysis is an approach to better understand the general biological processes that are affected when a gene set shows differential expression in the condition studied. Applying this approach using the predicted miRNA targets could indicate if particular biological processes and gene pathways were likely controlled by miRNA guided post-transcriptional regulation. Therefore, gene enrichment analysis was performed with the predicted targets. This analysis revealed many enriched biological processes and gene pathways that were previously reported as smoltification related. Functional groups of lipid homeostasis, lipid synthesis and cholesterol biosynthesis (Figure 2 and Figure 3) were among those observed as enriched. In addition, regulation of lipoprotein lipase activity and plasma lipoprotein remodeling was also enriched. Enriched lipid metabolism related biological processes are in agreement with previous studies reporting that lipid synthesis and liver glycogen (a major storage form of energy) decrease during smoltification [13,16]. Changes in lipid metabolism may affect tissue fatty acid compositions as previous study suggested that fatty acid compositions changed as a pre-adaptive response to seawater entry [77,78]. Functional groups related to energy metabolism including amino acid catabolic process, fatty acid metabolic process and acyl-CoA biosynthetic process (Figure 2 and Figure 3) were also enriched. Acyl-CoA enters the citric acid cycle in the mitochondrion by combining with oxaloacetate to form citrate catalyzed by citrate synthase [16,79]. Subsequent oxidation of citrate provides the major source of cellular ATP production which is essential in the smoltification as it is a highly energy-demanding process [80]. The enrichment of the functional groups related to energy metabolism observed shows that miRNAs may fine tune the genes important in energy metabolism which is essential to success in this developmental transition [16,18,20].

Other enriched biological processes and pathways that were specifically related to smoltification were metabolism of proteins and amino acids (e.g., cytosolic tRNA aminoacylation, cellular amino acid catabolic process, metabolism of amino acids and derivatives). The contents of free amino acids have previously been reported to reorganize during smoltification. This was suggested to be linked to changes in cellular osmoregulatory gradients [17]. Glucuronidation which is an important detoxification process occurring in fish liver [59] was also enriched, indicating that miRNAs may involve in regulation of detoxification in liver during smoltification.

Another interesting enriched biological process was circadian rhythm, an endogenous time-keeping system that controls and coordinates metabolism, physiology and behavior to adapt to variations within the day and the seasonal environmental cycles [81]. This indicates that miRNAs may also regulate genes involved in circadian rhythm which is likely to be affected by changes in photoperiod during smoltification. Circadian rhythm is important for smoltification as they translate changes in daylength into physiological response [26].

Various signaling pathways related to signal transduction, processes that regulate overall growth and cellular proliferation, differentiation and survival [82] were also enriched among miRNA targets. The enriched signaling pathways observed here, e.g., signaling by TGF-beta receptor complex, NR1H2 and NR1H3 regulate gene expression linked to lipogenesis and signaling by MET, may play a crucial role in affecting cellular metabolism during smoltification and in the adaptation to seawater.

The results showed that gene pathways associated with immune system were enriched among the gDE-miRNA target genes. This indicates that miRNAs may be involved in the regulation of immune system changes during smoltification and adaptation to seawater. It has also been reported that decreased immune defenses to pathogens was correlated with the high energetic cost due to smoltification related changes [7,83]. Both of these biological processes are associated with the gDE-miRNAs by their targets being enriched in these groups. Biological processes related to stress response (e.g., cellular response to oxidative stress, response to endoplasmic reticulum stress and regulation of transcription from RNA pol II promoter in response to stress) as well as liver development and angiogenesis were enriched. Together, this shows that miRNAs may influence genes important in stress response and liver development associated with smoltification and adaptation to seawater.

Interestingly, regulation of Insulin-like Growth Factor (IGF) transport and uptake by Insulin-like Growth Factor Binding Proteins (IGFBPs) was also enriched. Previous studies reported that growth hormone (GH) increased during smoltification [84] and GH stimulates IGF-1 and IGF-2, leading to modulation of intermediary metabolism and osmoregulatory mechanism in fish [85,86]. Furthermore, the interaction of cortisol with GH, IGF-1 and corticosteroid receptors promotes salinity tolerance, changes in growth and metabolism [26]. The enrichment of these groups indicates that miRNAs may be involved in the IGF-associated changes occurring during smoltification.

## 5. Conclusions

In conclusion, enrichment analysis of the predicted target genes revealed that they were significantly enriched in important biological processes groups and pathways associated with smoltification and adaptation to seawater. Whether any of the gDE-miRNAs are themselves among molecular initiators of the smoltification process or they regulate the downstream cellular processes triggered by this developmental transition, is uncertain. Nevertheless, this approach allowed us to identify miRNAs associated with smoltification and predict high confident target genes. The findings in this study pave the way for validation of the predicted target genes and further studies of such miRNA–mRNA interactions by experimental assays.

## Figures and Tables

**Table 1:** Photoperiod and water temperature during the experimental trial.

Experimental Days	Hours of Light per Day (h)	Water Temperature (°C)	Water Type
Day 0	24	8	Fresh water
Day 1–5	12	13	Fresh water
Day 6–47	12	12	Fresh water
Day 48–60	24	12	Fresh water
Day 61–81	24	8	Fresh water
Day 82–111	24	8	seawater

**Table 2:** Time-points and conditions where liver samples were collected.

Group	Sample Collection Time Points	Light ^1^	Temp. ^2^	Weight ^3^	Water Type	Sampling ^4^
T1	Parr, 1 day prior to light treatment	24	8	29.4 ± 5.6	Fresh water	Day 0
T2	Halfway into light treatment	12	12	52.6 ± 5.9	Fresh water	Day 47
T3	Three quarters into light treatment	24	8	63.9 ± 10.1	Fresh water	Day 67
T4	Smolt, 1 day prior to SWT	24	8	72.4 ± 8.7	Fresh water	Day 81
T5	One week after SWT	24	8	63.2 ± 8.5	Seawater	Day 88
T6	One month after SWT	24	8	98.4 ± 14.9	Seawater	Day 111

^1^ Hours with day light. ^2^ Water temperature in degree Celsius (°C). ^3^ Average weight in gram of the experimental fish collected at each time points. ^4^ Sampling day within the experimental period.

**Table 3:** Overview of differentially expressed guide miRNAs in cluster 1, 2 and 3.

Cluster 1.	Cluster 2	Cluster 3
ssa-miR-1-3p	ssa-let-7e-5p	ssa-let-7b-3p
ssa-miR-15d-5p	ssa-miR-19a-2-3p	ssa-let-7j-3p
ssa-miR-15e-5p	ssa-miR-29b-1-5p	ssa-miR-15a-3p
ssa-miR-17-5p	ssa-miR-93a-5p	ssa-miR-15bf-5p
ssa-miR-18bc-5p	ssa-miR-93b-5p	ssa-miR-15e-3p
ssa-miR-20a-5p ^2^	ssa-miR-101b-3p ^2^	ssa-miR-30a-1-2-5p
ssa-miR-101a-3p ^1^	ssa-miR-146a-5p	ssa-miR-30a-3-4-5p
ssa-miR-106a-5p	ssa-miR-146b-5p	ssa-miR-30e-5p
ssa-miR-106b-5p	ssa-miR-200b-3p	ssa-miR-122-1-3p
ssa-miR-125b-1-3p	ssa-miR-200c-3p	ssa-miR-122-2-3p ^1,2^
ssa-miR-130a-2-3p	ssa-miR-200d-3p	ssa-miR-203b-3p
ssa-miR-142b-5p	ssa-miR-218a-5p	ssa-miR-205b-5p
ssa-miR-146d-5p	ssa-miR-218b-5p	ssa-miR-206-3p
ssa-miR-153a-3p ^2^	ssa-miR-221-3p	ssa-miR-210-1-5p
ssa-miR-216c-3p	ssa-miR-222a-5p	ssa-miR-214-1-2-3p
ssa-miR-462b-5p	ssa-miR-222b-3p	ssa-miR-214-3-3p
ssa-miR-551a-3p	ssa-miR-novel-16-5p	ssa-miR-301a-5p
ssa-miR-551b-3p		ssa-miR-455-3p
		ssa-miR-457ab-5p ^2^
		ssa-miR-8163-3p
		ssa-miR-8163-5p ^1,2^
		ssa-miR-novel-1-3p
		ssa-miR-novel-2-5p
		ssa-miR-novel-10-3p
		ssa-miR-novel-12-5p
		ssa-miR-novel-13-5p
		ssa-miR-novel-16-3p ^2^

The annotation (^1^) indicates liver-specific miRNAs and (^2^) indicates miRNAs with large changes from T1 to T4.

## Data Availability

All sequenced samples have been submitted to the NCBI Sequence Read Archive Centre (SRA) (https://www.ncbi.nlm.nih.gov/sra accessed 20 April 2022) with accession bioproject number PRJNA665200.

## Supplementary Materials

The following are available online at https://www.mdpi.com/article/10.3390/biology11050688/s1, Figure S1: Heatmap of 88 DE-miRNAs, Table S1: Overview of the 42 experimental liver samples, Table S2: DE-miRNAs with their family, mature sequences and relative expression values, Table S3: Complete overview of DE-miRNAs and their corresponding 3p or 5p mature sequences, Table S4: Overview of differentially expressed mRNAs, Table S5: Overview of enriched DE-mRNAs, Table S6: List of all predicted targets of guide DE-miRNAs, Table S7: List of unique gene symbols from DE-miRNAs predicted target genes, Table S8: List of enriched GO biological processes with fold enrichment over 2, Table S9: List of enriched pathways with fold enrichment over 2.
