# Supplementary material for: Expression Analysis in Atlantic Salmon Liver Reveals miRNAs Associated with Smoltification and Seawater Adaptation
Source: Biology (Basel). 2022 Apr 30;11(5):688. doi: 10.3390/biology11050688 (PMC9138835; doi:10.3390/biology11050688)
Supplement: Supplementary file 1 [file biology-11-00688-s001.zip › Figure S1. Heatmap of 88 DE-miRNAs.pdf]

Heatmap and hierarchical clustering of the 88 differentially expressed miRNAs

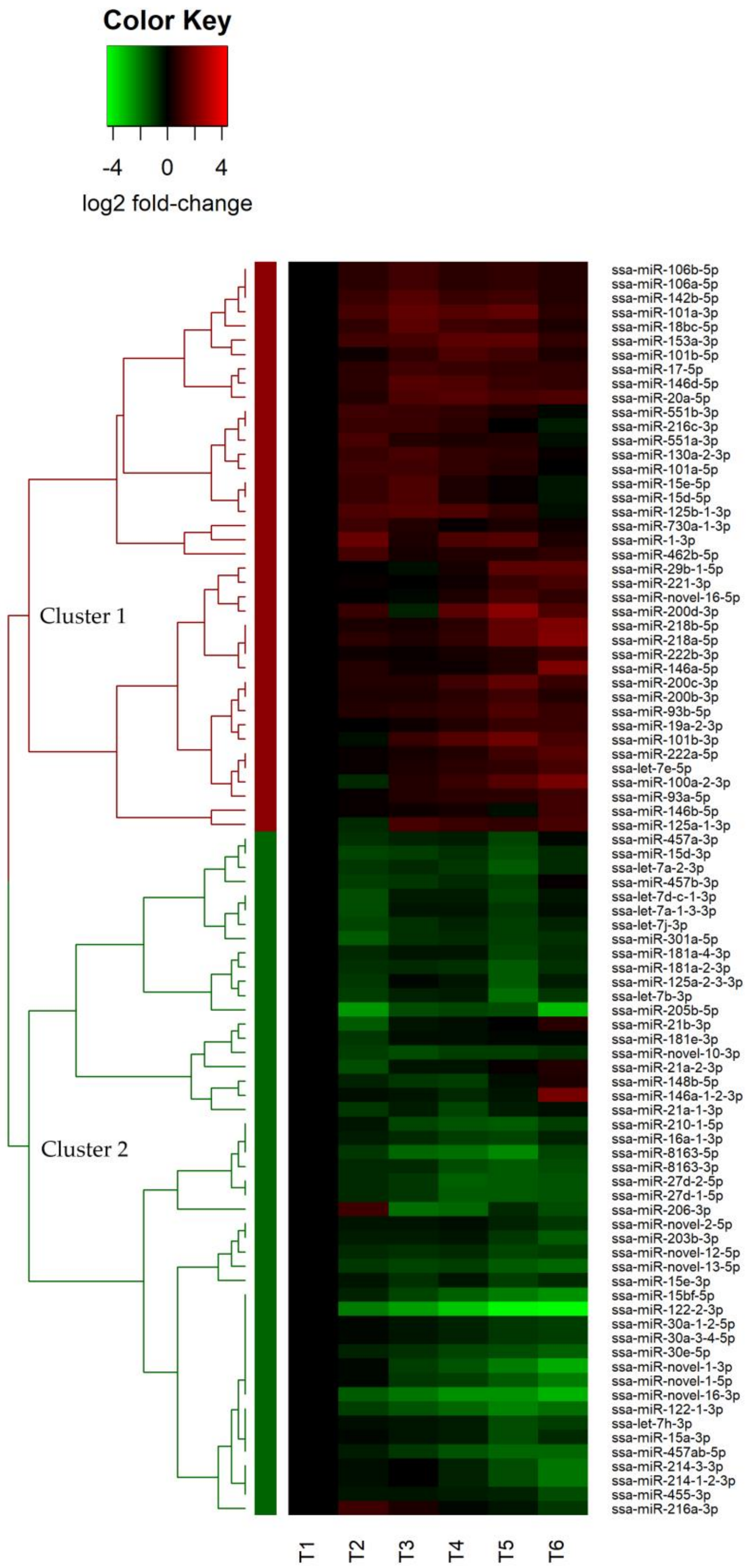

Figure S1. Heatmap and hierarchical clustering of the 88 differentially expressed miRNAs (DE-miRNAs). Each row represents a miRNA and each columns represents the expression changes at each time points relative to T1 (pre-smolt, one day before smoltification). T2-T4 and T5-T6 are relative expression changes during smoltification period and post SWT period, respectively. The dendrogram and the row side colours on the left show the two major clusters of DE-miRNAs (Cluster 1 – red and Cluster 2 – green).
